# Issues in applying multi-arm multi-stage methodology to a clinical trial in prostate cancer: the MRC STAMPEDE trial

**DOI:** 10.1186/1745-6215-10-39

**Published:** 2009-06-11

**Authors:** Matthew R Sydes, Mahesh KB Parmar, Nicholas D James, Noel W Clarke, David P Dearnaley, Malcolm D Mason, Rachel C Morgan, Karen Sanders, Patrick Royston

**Affiliations:** 1 MRC Clinical Trials Unit, London, UK; 2 Queen Elizabeth Medical Centre, Birmingham, UK; 3 Christie and Salford Royal Foundation Trusts, Manchester, UK; 4 Institute of Cancer Research and Royal Marsden Hospitals, Sutton, UK; 5 Cardiff University School of Medicine, Cardiff, Wales, UK

## Abstract

**Background:**

The multi-arm multi-stage (MAMS) trial is a new paradigm for conducting randomised controlled trials that allows the simultaneous assessment of a number of research treatments against a single control arm. MAMS trials provide earlier answers and are potentially more cost-effective than a series of traditionally designed trials. Prostate cancer is the most common tumour in men and there is a need to improve outcomes for men with hormone-sensitive, advanced disease as quickly as possible. The MAMS design will potentially facilitate evaluation and testing of new therapies in this and other diseases.

**Methods:**

STAMPEDE is an open-label, 5-stage, 6-arm randomised controlled trial using MAMS methodology for men with prostate cancer. It is the first trial of this design to use multiple arms and stages synchronously.

**Results:**

The practical and statistical issues faced by STAMPEDE in implementing MAMS methodology are discussed and contrasted with those for traditional trials. These issues include the choice of intermediate and final outcome measures, sample size calculations and the impact of varying the assumptions, the process for moving between trial stages, stopping accrual to each trial arm and overall, and issues around perceived trial complexity.

**Conclusion:**

It is possible to use the MAMS design to initiate and undertake large scale cancer trials. The results from STAMPEDE will not be known for some years but the lessons learned from running a MAMS trial are shared in the hope that other researchers will use this exciting and efficient method to perform further randomised controlled trials.

**Trial registration:**

ISRCTN78818544, NCT00268476

## Background

### Rationale for MAMS trials

The multi-arm, multi-stage (MAMS) design[1] is a new efficient, adaptive approach for conducting randomised controlled trials. It allows several agents or combinations of agents to be assessed simultaneously against a single control group in a randomised controlled trial (RCT). Recruitment to research arms that do not show sufficient promise in terms of an intermediate outcome measure may be discontinued. By contrast, recruitment to the control arm and to promising research arms continues until sufficient numbers of patients have been entered to assess the impact in terms of the definitive primary outcome measure.

By assessing several treatments in one trial, the MAMS design allows reliable information on the value of the treatments to be acquired more quickly and with smaller numbers of patients compared with a program of separate Phase II and Phase III trials. This adaptive design, one of a number of seamless Phase II/III designs [2], allows continuing investment to be focused on treatments that show promise, whilst discontinuing investigation of therapy with insufficient evidence of activity.

Initiating clinical trials, particularly in the current regulatory environment, is resource-intensive and time-consuming. The MAMS design provides a cost-efficient method requiring fewer research approvals. The method increases the likelihood that a single trial will identify a successful treatment and it decreases the likelihood that the whole trial would have to be stopped prematurely, as the chance that all treatment regimens are either insufficiently tolerated or ineffective is reduced if there are many arms. Furthermore, if all arms stop early, the trial has successfully closed off multiple avenues of research allowing other approaches to be examined more speedily. Further detail on the general rationale for the MAMS design has been published elsewhere[3].

### Rationale for STAMPEDE

**Figure 1:**
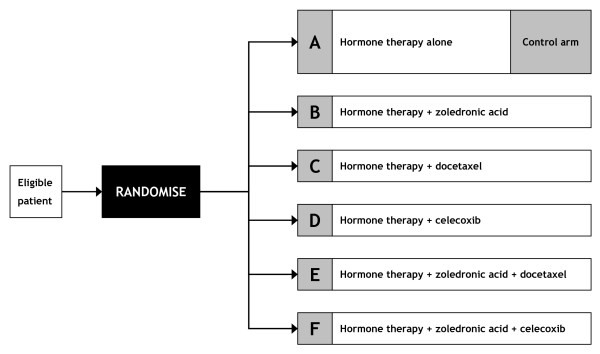
**STAMPEDE trial arms**. The randomisation ratio is 2A : 1B : 1C : 1D : 1E : 1F. HT = hormone therapy, *bid *= twice daily.

STAMPEDE (MRC PR08, ISRCTN78818544, NCT00268476) is a large, multi-centre, open-label, MAMS (6-arm, 5-stage) RCT for men with high-risk localised or metastatic prostate cancer who are being treated for the first time with long-term hormone therapy, also known as androgen deprivation therapy (ADT) or androgen suppression. The trial assesses five new strategies, adding treatments to the current standard hormone therapy in this patient group, testing whether: (i) two intravenous drugs (docetaxel and zoledronic acid) currently used in the later stages of disease can have a beneficial effect when given earlier; (ii) an orally-administered cox-2 inhibitor (celecoxib) can impact high-risk prostate cancer; and (iii) combinations of these agents that have shown promise *in vitro *will produce patient benefit. The design of the trial is summarised in Figure 1. The detailed clinical rationale of the trial has been published elsewhere [4-6] and further information is available on the trial website [7].

### Rationale for publication

STAMPEDE is the first MAMS trial to implement multiple arms and multiple stages synchronously. The purpose of this paper is to describe the methodology of the STAMPEDE trial in order to illustrate and discuss the MAMS trial design and implementation in to practice. It focuses on the issues we have tackled in developing STAMPEDE, revealing the choices and compromises that have been made to date. These encompass issues in design and conduct such as the weighted randomisation ratio, the use of one-sided tests, why a factorial design is not used and how the individual arms or the trial overall may be stopped early for lack-of-efficacy, for efficacy or for safety.

## Issues in outcome measures and target difference

### Choosing the primary outcome measures

Key to any clinical trial is the choice of outcome measures (OM). The MAMS design requires definitive primary and intermediate primary OM. The definitive OM is the one upon which final conclusions should be based while the intermediate OM provides a means of screening for emerging evidence of activity. The intermediate OM need not be a true surrogate[8] for the definitive OM, but it is assumed that the intermediate OM occurs earlier and more frequently than the definitive OM and that it is on the causal pathway. In some situations, the intermediate OM may be the same as the definitive OM e.g. if both are overall survival but one represents an earlier review.

In STAMPEDE, the intermediate and definitive OM are different and so the intermediate stages are deemed "Activity Stages"; the final stage is deemed the "Efficacy Stage". Table 1 presents the primary OMs at each trial stage. Overall survival (OS) was chosen as the definitive outcome measure in STAMPEDE. The additional time required to follow-up for maturity of this outcome is outweighed by its objective, verifiable nature and its clear relevance to this high-risk patient population. We have chosen failure-free survival (FFS) as the intermediate outcome measure, defined as progression of, or death from, prostate cancer. "Progression" includes biochemical failure, measured by serum prostate specific antigen (PSA), a marker used in assessing prostate cancer control.

**Table 1:** Trial Outcome Measures

**Trials stage**	**Primary outcome measures**	**Secondary outcome measures**
**Pilot phase**	Safety*	Feasibility
		
**Activity Stage I**	I: Failure-free survival (FFS)^†^	Overall survival (OS)
		Toxicity
		Skeletal related events
		
**Activity Stage II**	I: Failure-free survival (FFS)^†^	Overall survival (OS)
		Toxicity
		Skeletal related events
		
**Activity Stage III**	I: Failure-free survival (FFS)^†^	Overall survival (OS)
		Toxicity
		Skeletal related events
		
**Efficacy Stage IV**	D: Overall survival	Quality of life
		Cost effectiveness
		Failure-free survival^†^
		Toxicity
		Skeletal related events

*Based on toxicity

^†^Including biochemical failure (see protocol)

The assumption in using the MAMS design is that if the null hypothesis of no difference is true for the intermediate OM, it must be true for the definitive OM. It is not necessary that a true alternative hypothesis for the intermediate OM translate into a true alternative hypothesis for the definitive OM, but a true alternative hypothesis for the definitive OM must imply a true alternative hypothesis for the intermediate OM. If not, this would question whether the treatment was acting on the disease or via a different pathophysiological mechanism. The size of the effects of treatment on the intermediate OM and definitive OM do not need to be highly correlated, although in practice they often will be.

In STAMPEDE, many more FFS events are anticipated than deaths. FFS is not strictly a surrogate for prostate cancer survival[9] but an improvement in FFS would be expected if there were to be an improvement in overall survival and, if there was little or no effect on FFS, then little or no effect on OS would be anticipated. We accept that some patients who join the trial will die without disease progression from causes other than prostate cancer or its treatment; correspondingly, some patients will experience a FFS event but die, ultimately, from other causes.

### Choosing the target difference for the definitive primary outcome measure

STAMPEDE is targeting a hazard ratio (HR) of 0·75 for OS for each of the five research arms over the control arm: a 25% relative decrease in risk. If the median survival time were five years, this would translate into an absolute improvement of 10% (from 50% alive to 60% at five years). This is a realistic benefit to target: the hazard ratios seen for docetaxel in two RCTs for men with hormone-refractory prostate cancer were 0·76 (95% confidence interval (CI) 0·62 to 0·94)[10] and 0·80 (95% CI 0·67 to 0·97)[11]; the hazard ratio reported, thus far, for zoledronic acid in the early breast cancer setting is 0·60 (95% CI 0·32 to 1·11)[12]. We are testing a number of treatment approaches with different profiles of toxicity, acceptability and cost which are currently not fully known. Against this uncertain background, it is anticipated that an absolute difference of 10% in 5-year survival would be large enough for these treatments to be considered worthwhile.

### Choosing the target difference for the intermediate outcome measure

STAMPEDE is also targeting a hazard ratio of 0·75 for FFS for each research arm over the control arm at each Activity Stage: the same effect size as for OS. Given adequate power for survival, this is perhaps conservative because it is reasonable to expect a larger treatment effect on FFS than OS. Recent trials for men with prostate cancer that have demonstrated statistically significant benefits on OS have reported larger effects on FFS[11,13,14]. This [15]has also been reported in other disease settings [16-19].

### Choosing the secondary outcome measures

The secondary outcome measures, as in any trial, help to express the full picture of relevant outcomes that will support or refute the primary outcome measure. Table 1 presents the secondary OMs at each trial stage. It can be noted that each primary outcome measure in a given stage is a secondary outcome measure in another stage ie the various outcomes are simply emphasised differently over time.

## Issues in design and sample size

### Choosing the number of MAMS stages

The choice of four MAMS stages for STAMPEDE was a pragmatic one with three intermediate Activity Stages and one Efficacy Stage. The analyses need to be separated in time such that meaningful amounts of new information can be accumulated between reviews. The overall trial duration (accrual plus follow-up) was estimated to be about 7 years. We planned three intermediate reviews starting after the first 110 FFS events in the control arm with subsequent reviews planned with at least 100 further control arm FFS events between reviews. At each timepoint, the activity of each research arm, together with the safety data, is reviewed by the Independent Data Monitoring Committee (IDMC). Of course, the precise timing of the intermediate reviews depends on the observed accrual and event rates. The IDMC will meet at least annually throughout the trial to review safety data but the timing of meetings is sufficiently flexible to allow review at the appropriate times.

We also wished to undertake an early assessment of the safety of the research arms, particularly the combination treatments and the feasibility of accrual. Therefore, we defined a safety analysis to take place 6 months after recruitment to an additional Pilot Phase (a fifth stage) had been completed: when approximately 30 patients on each research arm had been in the trial for around 18 weeks. Any recommendations by the IDMC on the future conduct of the trial at this first stage were to be based solely on early safety signals. Importantly, this design allowed for the smooth transition of the trial from its 'safety and feasibility' phase, through to its first activity stage, without the need to set up a new trial.

### Choosing the size of the intermediate activity "hurdles"

The MAMS design determines a stopping guideline based on *lack of sufficient *activity (early stopping for activity is discussed later). By considering the null and alternative hypotheses, significance level and power, we set, for each intermediate review, a critical value, also called a critical cut-point or "hurdle". Our null hypothesis is that there is no difference between a given research arm and the control arm; the alternative hypothesis is that there is a difference. At all intermediate stages, we require high power, whereas the significance level becomes increasingly strict with each stage. If the null hypothesis of no difference is rejected at a particular stage, the treatment arm passes to the next stage where greater evidence is required to reject the same null hypothesis.

### Calculating the number of patients required

The concept of a single recruitment target is not appropriate for like STAMPEDE because there are many factors that may affect the accrual target. Like a traditional RCT with a time-to-event primary outcome measure, the sample size for STAMPEDE is based on events rather than patients. For design purposes, we assume a constant hazard rate in each arm over time i.e. exponential survival. The required number of events is based on set factors including the randomisation ratio and the expected (target) difference between the research and control arms. The power and significance level are set for each intermediate (activity) stage. Like all trials, we need to estimate some factors that may be more difficult to predict, such as the expected event and recruitment rates. The final unpredictable factor is the number of arms recruiting at each stage.

By specifying these variables, the number of control arm events required for each stage can be calculated (Table 2). We discuss the setting of these design parameters, below, in a scenario that requires 3,100 patients.

**Table 2:** Guidelines for stopping accrual to the i^th ^research arm at intermediate analyses

**Activity Stage**	**Timing of analysis: control arm events**	**Critical value** **(Hazard Ratio)**
I	114	1·00
II	215	0·92
III	334	0·89

The intermediate analyses are timed according to the number of events reports on the control arm. Discontinuation of recruitment to a research arm will be considered if the observed hazard ratio is greater than the critical value (also known as the critical cutpoint or "hurdle")

#### Power

We would not wish to discard any treatment which has a true effect that is at least as good as the effect targeted in the alternative hypotheses. Thus, we require a high level of power at all stages, particularly at Activity Stages I-III. For STAMPEDE, 95% power (β = 0·05) has been chosen. At Efficacy Stage IV, this can be relaxed, perhaps, to more traditional values and 90% power (β = 0·10) has been chosen. The overall power across the trial is approximately 85%, although the exact value is dependent on the correlation between the treatment effects on the FFS and OS outcome measures[1]. The lower limit of overall power could only be as low as 77% and then only if the treatment effects at all of the stages are independent, a very unlikely scenario.

#### Significance level

To allow us to retain high power at all times and still conduct intermediate analyses during the recruitment, we have relaxed the significance level for tests, allowing informative statements to be made as early as possible. We accept that, inevitably, this will be with some error. This error is, however, a conservative one, allowing treatments to pass to the next stage inappropriately. STAMPEDE employs one-sided tests throughout the activity stages because our interest only lies in identifying signals that a research regimen is better. We chose a one-sided significance level of 0·50 for Activity Stage I, 0·25 for Activity Stage II, 0·10 for Activity Stage III and 0·025 for Efficacy Stage IV. The overall type I error across the four Activity/Efficacy Stages for the pairwise comparisons of research arm against control is calculated as approximately 0·013, although the exact value is dependent on the correlation of the treatment effect on the intermediate and final outcome measures.

#### Event rates

STAMPEDE includes both patients with and without metastatic disease. Median FFS is estimated at 2 years and median OS at 4 years, estimated from published data[20] in men with metastatic disease[21] and castrate refractory disease[22].

#### Allocation ratio

The allocation ratio is set at 2 patients allocated to the control arm for 1 patient allocated to each of the research arms i.e. 2:1:1:1:1:1. This is biased towards the control arm because this arm is the comparator for each research arms and as good as possible an estimate of control arm event rate is required. It can be shown empirically that, for a fixed number of patients randomised and given the other parameters used in STAMPEDE, this allocation ratio maximises the power for each pairwise comparison and for a fixed power reduces the time-to-maturity (trial duration).

#### Accrual

Accrual is assumed at a constant rate of 500 patients per year, based on an estimate of >40 patients per month joining the trial when all sites are open across the UK.

#### Arms recruiting

This reference scenario presumes that one arm will stop recruitment after each intermediate review of activity. This cannot be accurately predicted in advance; as many arms as necessary will be permitted to continue recruitment in future stages.

### Number of patients required for STAMPEDE

**Figure 2:**
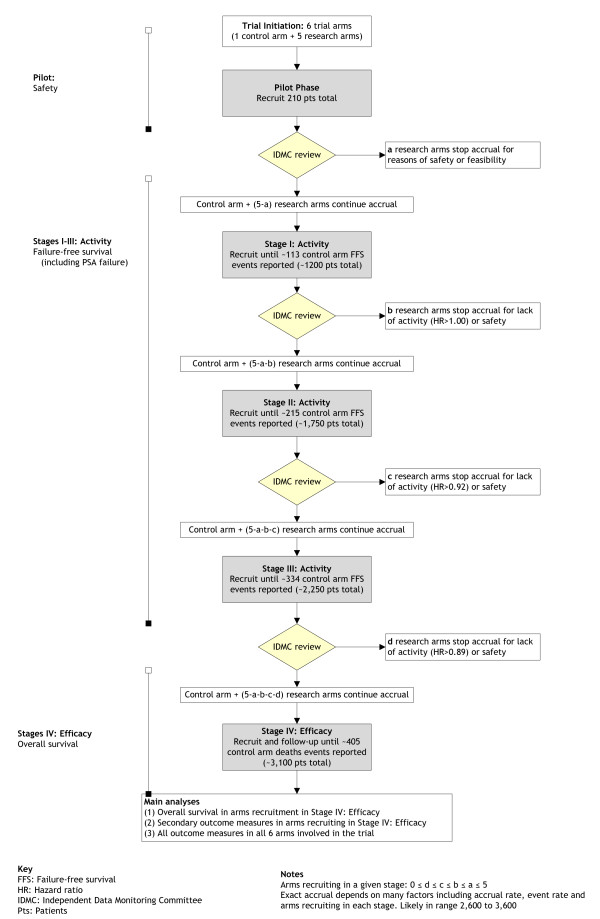
**Progress of STAMPEDE through the trial stages**.

**Figure 3:**
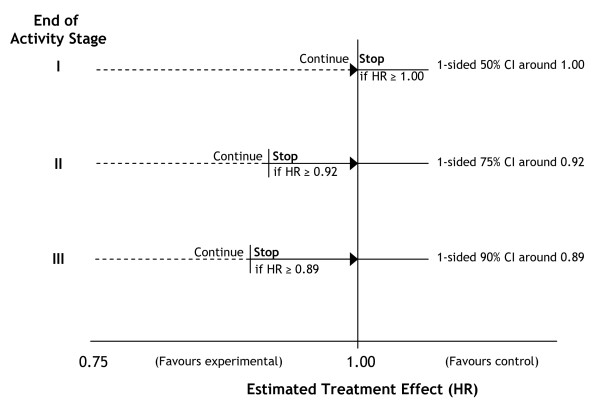
**Hazard ratio cutpoints for intermediate reviews**. HR = Hazard ratio; CI = Confidence interval.

Figure 2 presents a flow diagram for STAMPEDE depicting the reviews and the potential discontinuation of further recruitment to research arms. The critical values are detailed in Table 2 and presented graphically in Figure 3. For the second intermediate review of activity data, for example, the critical value is set at HR = 0·92 (Figure 3). This analysis will take place when around 216 failure-free survival events have been reported in patients allocated to the control arm. If the point estimate of the HR for a given research arm is 0·92 or lower we would reject, using a significance level of 0·25, an intermediate null hypothesis that there is no difference between the control arm and this research arm. We can also see, therefore, a one-sided 75% confidence interval would exclude HR = 1·00, the null hypothesis. If the null hypothesis for this arm were rejected at end of Activity Stage II, recruitment to this arm continues in Activity Stage III. If the null hypothesis were not rejected (estimated HR>0·92), we would conclude that there was insufficient evidence of activity to justify continuing recruitment to these arms. Follow-up of patients randomised previously would continue and the arm would be included in the final analyses (see below).

### Software for calculating sample size and critical values

**Figure 4:**
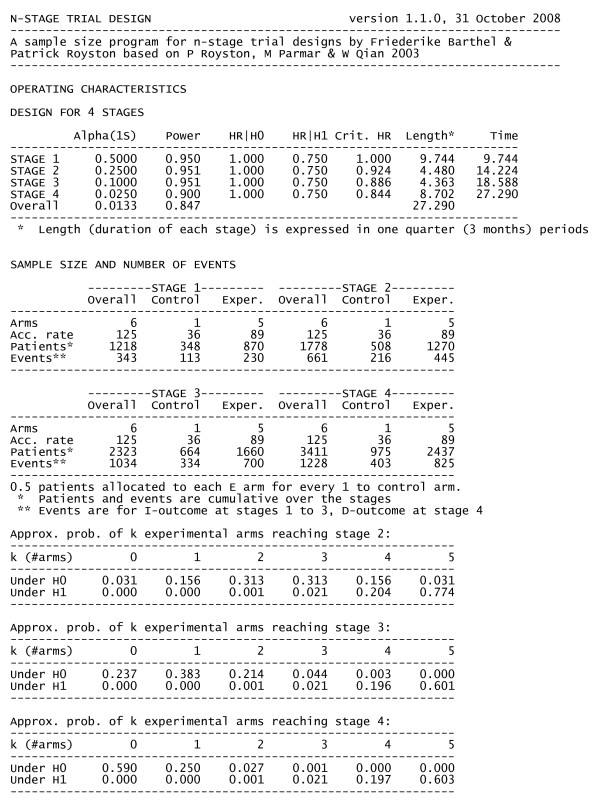
**Example output from -nstage- for the reference scenario**. This figure shows the exact output from using -nstage-. This does not include the Pilot stage for safety, concentrating only the particular issues relating to the application of the MAMS activity and efficacy stages. In this example, the durations are expressed in quarter-years. The variable factors have been chosen such that no arms are stopped early for lack-of-efficacy (there is 1 control arm and 5 research arms in each stage); the accrual rate is set at 500 patients/year; the median progression-free survival (PFS) and overall survival are estimated to be 24 months and 48 months, respectively; and accrual is uncapped ie recruitment continues to the point of overall maturity. The power was set at 95% for the three activity stages and the observed values are consistent with this.

Construction of the intermediate critical values is not a simple process and manual calculation is not recommended. STAMPEDE uses a freely-available sample size program for designing MAMS trials, developed for Stata (College Station, TX, USA) called nstage (Barthel FMS, Royston P, Parmar MKB: A menu-driven facility for sample size calculation in novel multi-arm, multi-stage randomised controlled trials with a survival-time outcome, submitted). Figure 4 presents, as one example, the output for a reference scenario where no arms stop accrual early.

### Impact of changing the variable parameters

It is difficult to predict the number of patients required for STAMPEDE because of the many variable parameters. We have performed sensitivity analyses using nstage to examine the impact on target accrual and trial duration of changing some parameters, whilst keeping the target difference, allocation ratio, significance level and power constant.

At the time the trial was opened, there was uncertainty about the willingness of doctors and patients to support a trial with 6 arms and the ability or necessity to recruit outside the UK was uncertain. Therefore, scenarios were calculated based on constant accrual rates of 350, 500 and 750 men per year (although nstage permits different accrual rates in each stage).

The trial has broad eligibility criteria, being aimed at men starting long-term hormone therapy for the first time. At the outset there was uncertainty regarding the likely disease stage distribution in men with this spectrum of high-risk prostate cancer. The lower the proportion with metastatic disease (and, conversely, the higher the proportion with high-risk localised disease), the lower the event rate. Many doctors believed they saw fewer men with newly-diagnosed hormone-sensitive metastatic disease in current practice than previously. However, ongoing accrual to STAMPEDE shows that this patient group remains large. We also assumed that median OS time would be double the median FFS time, but with the increasing use in standard practice of chemotherapy with docetaxel at relapse[10,11], we have subsequently considered whether survival might be proportionately longer[23]. Scenarios were, therefore, calculated based on median FFS/OS, respectively, of (i) 18/36, (ii) 24/48, (iii) 30/60 and (iv) 24/60 months for patients on the control arm.

These sensitivity analyses culminated in the production of a "Statistical Design Document", including the presentation of over 400 combinations of the variables described above; Table 3 is a small extract which summarises the impact of altering any one of these factors independently. In these examples, the reference scenario assumes that no research arms stop recruitment early. For example, it can be seen that slowing annual accrual by 150 patients from 500 to 350 patients per year decreases the accrual target from 3,411 to 2,960 but lengthens the trial duration from 82 months to 102 months. Conversely, increasing annual accrual by 250 patients from 500 to 750 patients per year increases the accrual target from 3,411 to 4,046 but shortens the trial duration from 82 months to 65 months. A 6-month increase in median FFS in the control arm from 24 to 30 months increases the trial duration by 8 months; decreasing from 24 to 18 months shortens the trial duration by 9 months. Finally, if recruitment to all bar one of the research arms is stopped after the first intermediate activity analysis, trial duration shortens by 16 months. Thus, sensitivity analyses show that altering these parameters can have a substantial impact on the total number of patients required and on the overall duration of the trial.

**Table 3:** Impact of variable factors on STAMPEDE target accrual and duration

	**Median**		**Arms accruing at stage**				**Total**		**Differences in**	
**Pts/yr**	**Median FFS (m)**	**Median OS (m)**	**1**	**2**	**3**	**4**	**Pts**	**Time (m)**	**Total pts**	**Time (m)**
**Reference**										
500	24	48	6	6	6	6	3411	82	0	0

**Impact of accrual rate**										
350	24	48	6	6	6	6	2960	102	-451	20
500	24	48	6	6	6	6	3411	82	0	0
750	24	48	6	6	6	6	4046	65	635	-17

**Impact of FFS event rates**										
500	18	36	6	6	6	6	3040	73	-371	-9
500	24	48	6	6	6	6	3411	82	0	0
500	30	60	6	6	6	6	3743	90	332	8

**Impact of OS event rates**										
500	24	48	6	6	6	6	3411	82	0	0
500	24	60	6	6	6	6	3743	90	332	8

**Impact of dropping arms**										
500	24	48	6	6	6	6	3411	82	0	0
500	24	48	6	6	6	2	3190	77	-221	-5
500	24	48	6	6	2	2	2983	72	-428	-10
500	24	48	6	2	2	2	2738	66	-673	-16
500	24	48	6	5	4	3	3133	75	-278	-7

Difference = difference from reference scenario, FFS = failure-free survival, OS = overall survival, Pts = patients, M = months. This does not include the Pilot stage for safety, concentrating only the particular issues relating to the application of the MAMS activity and efficacy stages

The first intermediate reviews for activity (end of Activity Stage I) are expected to take place late in 2009. At this point, we will have a better indication of the accrual rates in the longer term, the FFS event rates and the number of arms that will continue recruitment into Activity Stage II; this will facilitate better estimation of the number of patients that will actually be required.

### How the number of statistical tests affects the calculations

The MAMS design means that recruitment to arms may be stopped due to a lack of benefit. In this setting, we wish to protect against false negatives: issues of power are more important than the significance level. Since there are five research arms, there are five principal comparisons at Activity Stage I. However, issues of multiple testing are not a major source of concern at this stage as we are stopping for lack of benefit; it is more important in Activity Stages I-III to maintain high power so a truly effective regimen can continue to Efficacy Stage IV.

## Issues at the end of a MAMS stage

### The end of an Activity Stage

The intermediate analyses for a given Activity Stage take place when a pre-specified number of events in the control arm have been reported. The safety and activity data are then presented to the IDMC. Even with a perfectly timed process, it is unrealistic to expect this to occur instantly. Whilst these analyses are performed and are being reviewed, trial recruitment continues as for most other trial with interim analyses.

The IDMC will meet to review the accumulating trial data in the context of any relevant external data and make recommendations to the trial's executive body, the Trial Steering Committee (TSC), who will then make a decision. The TSC may recommend stopping accrual to one or more arms, or to none, but the issues of managing this process are not specific to MAMS trials. The MAMS stopping guideline *is j*ust a guideline. It is possible that the IDMC, upon seeing intermediate data, may recommend a research arm stop accrual despite being on the favourable side of the intermediate critical value; or it may continue accrual despite being on the unfavourable side (see below).

### Managing the stopping of an arm

In practice, if an arm is stopped early some information will have to be shared with many parties. If accrual stops for reasons of safety, the action required is not specific to STAMPEDE or MAMS trials: the termination of trial treatment and the appropriate assessment of patients will be carried out as for a traditionally-designed trial.

If accrual to an arm is stopped for insufficient activity after an intermediate review, assessment regarding treatment cessation or continuation will need to be considered in those patients still receiving the therapy. For example, it is possible that accrual to an arm will be stopped because it is showing limited but insufficient activity, rather than because it is harmful. In this case, comprehensive discussion will be required with the IDMC and subsequently the TSC and TMG, to agree recommendations about the continuation or discontinuation of trial treatment in these patients, and their cross-over to other trial regimens. This would likely involve the release of some unblinded information to the TSC.

Researchers may assume that arms that continue accrual after an intermediate review are showing some evidence of worthwhile activity. The intermediate data would not, however, be routinely released for scrutiny outside the IDMC. Familiarity with the MAMS design will help researchers to understand that the null hypothesis for the continuing arms has probably been rejected for a given level of evidence and that the next Activity Stage will assess the specific treatment arm more stringently. It would be wrong for researchers to be taken out of equipoise by this implicit intermediate information; instead, it should reinforce the need to continue with active randomisation to gain stronger evidence. Crucially, the intermediate assessments require only modest levels of evidence to continue accrual on an outcome measure which considers activity and not efficacy. This point will be particularly emphasised to investigators.

### How stopping accrual to one arm may impact on the other arms

If each of the trial arms contained different agents, the act of stopping accrual to one arm should not affect any of the others. However, STAMPEDE includes combinations of 3 research drugs across 5 research arms, so we must also think in terms of drugs as well as arms: stopping accrual to one research arm may impact on other trial arms, depending on the reason for cessation. If the results indicated that one research arm needed to be stopped for safety reasons, the safety data for any other arm(s) containing that drug would need careful consideration; the strength of the safety signal would need consideration in the context of emerging activity data.

**Figure 5:**
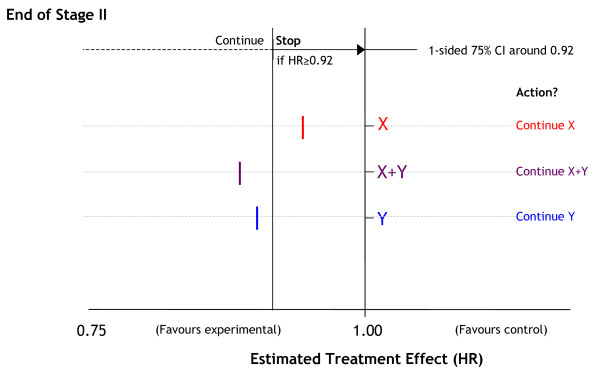
**Hypothetical intermediate results at the end of an intermediate trial stage**. HR = Hazard ratio; CI = Confidence interval. In this example, the research arms are drug A, drug B and the combination of drugs A and B; the common control arm is neither drug. The guideline for continuation of recruitment into the next stage is 0·92 compared with the control arm. The research arms containing drug B and drug A+B have met the criteria in this instance; the arm containing drug A has not met the criteria but is showing some evidence of an advantage in terms of the intermediate outcome measure. In this instance and in the absence of safety concerns, the IDMC may decide that recruitment should be continued to all 3 arms.

Intermediate critical values have been set for the research arms, but drugs should not be rejected inappropriately. Figure 5 presents a hypothetical 4-arm MAMS trial with two research drugs (X, Y) used alone or in combination and compared against a single control arm that contains neither X nor Y. In this example, the critical value for continuation of recruitment into the next stage is HR<0·92. The figure shows that both research arms containing drug Y have met the criteria and should continue recruitment in the next stage, providing there are no safety concerns. However, the arm containing only drug X has not met the criteria, although the point estimate of the HR is showing some evidence of activity. In this instance, and in the absence of safety concerns, it may be desirable to continue recruitment to all 3 arms unless there are convincing data that the benefit seen for the combination of drug X plus drug Y stems only from drug Y. Unless such data exist, it may be unwise to conclude, at the end of the trial, that X+Y is effective compared to control while X alone is ineffective compared to control. Continuing accrual to the X only arm facilitates eventual assessment of the relative contribution of X and Y to the X+Y combination.

### When overall recruitment is completed

The sample size calculations presented in the section "Calculating the number of patients required" assume that recruitment will continue to the point when the results are known. Of course, any man recruited just before overall trial recruitment stops will be very unlikely to contribute meaningfully to the final analyses; therefore, it is possible to discontinue recruitment promptly when it is clear that adequate numbers of events are achievable.

Table 4 shows the impact of stopping accrual after 5, 6 or 7 years. For example, if the number of arms recruiting is fewer than six after Activity Stage II, recruitment may be stopped earlier, after 5 or 6 years, without adversely affecting the trial duration. Recruitment to the trial would, therefore, be stopped earlier than is suggested in Table 3. At the start of last stage, Efficacy Stage IV, we will be able to obtain the best estimate of when the required number of control arm events will be observed and when accrual may best be stopped.

**Table 4:** Impact on patients recruited and trial duration of stopping accrual at a set timepoint

	**Median**		**Arms accruing at Activity Stage**					**Total**		**Differences^**b **^in**	
**Pts/yr**	**Median FFS (m)**	**Median OS (m)**	**1**	**2**	**3**	**4**	**Year accrual stopped**	**Pts**	**Time (m)**	**Total pts**	**Time (m)**
**No arms stop early**											
500	24	48	6	6	6	6	Uncapped^c^	3411	82	0	0
500	24	48	6	6	6	6	7	3411	82	0	0
500	24	48	6	6	6	6	6	3000	83	-411	1
500	24	48	6	6	6	6	5	2500	89	-911	7

**One arm is dropped after each Activity Stage**											
500	24	48	6	5	4	3	Uncapped^c^	3133	75	0	0
500	24	48	6	5	4	3	7	3133	75	0	0
500	24	48	6	5	4	3	6	3000	75	-133	0
500	24	48	6	5	4	3	5	2500	80	-633	5

**Dropped to 2 arms after Activity Stage I**											
500	24	48	6	2	2	2	Uncapped^c^	2738	66	0	0
500	24	48	6	2	2	2	7^c^	2738	66	0	0
500	24	48	6	2	2	2	6	2738	66	0	0
500	24	48	6	2	2	2	5	2500	66	-162	0

**Dropped to 2 arms after Activity Stage II**											
500	24	48	6	6	2	2	Uncapped^c^	2983	72	0	0
500	24	48	6	6	2	2	7^c^	2983	72	0	0
500	24	48	6	6	2	2	6	2983	72	0	0
500	24	48	6	6	2	2	5	2500	75	-483	3

**One arm dropped after each Activity Stage & survival is longer**											
500	24	60	6	5	4	3	Uncapped	3397	82	0	0
500	24	60	6	5	4	3	7^c^	3397	82	0	0
500	24	60	6	5	4	3	6	3000	83	-397	1
500	24	60	6	5	4	3	5	2500	91	897	91

**One arm is dropped after each Activity Stage & accrual is slower**											
350	24	48	6	5	4	3	Uncapped	2702	93	0	0
350	24	48	6	5	4	3	7	2450	94	-252	1
350	24	48	6	5	4	3	6	2100	101	-602	8
350	24	48	6	5	4	3	5^a^	*n/a*	*n/a*	*n/a*	*n/a*

Uncapped means that accrual continues until the point of maturity.

^a ^Model not provided because Efficacy Stage III would not have been completed by 5 years

^b ^Differences compared with the uncapped model with the same parameters

^c ^Recruitment would have been completed at sooner time point ie another model stops accrual sooner eg the first row shows uncapped accrual to be the same as accrual to 7 years because the accrual-to-7-years model stops accrual just before 7 years

## Issues in analysis

### Which arms are analysed at the end

The main analyses will be the separate, pairwise comparison of OS for research against control for each of the research arms still recruiting in Efficacy Stage IV. However, data from all randomised patients will contribute to analyses: all of the arms will be analysed at the end of the trial.

### If two or more arms are shown to be better than the control arm

If more than one research arm is better than the control arm, these research arms will be compared. Since this is a closed test procedure, there is protection of the type I error: two research arms would only be directly compared if *both *research arms are shown to be better than the control arm. There is limited power for any such comparison. However, such a comparison may have greater value than indirect comparison of these treatments from different trials. In the longer term, the appropriate action may be to maintain randomisation between these more beneficial research arms in a further period of accrual, as an extension of the MAMS design or as a new, separate trial that plans a combined analysis with longer-term results from STAMPEDE.

### Early stopping for efficacy

The MAMS stopping guidelines are set out formally for lack of benefit. The MAMS design does not provide specific guidelines for early stopping for sufficient evidence of efficacy, but may be supplemented with a traditional formal rule early for early stopping for efficacy, if required. The Haybittle-Peto rule[24], for example, would provide a structure for early stopping that does not affect the design parameters of a MAMS trial. Without using a formal stopping rule, early stopping may be possible using the commonly applied principle that the trial should report early only if the result would be convincing to a broad range of people, including those who were supportive of the research arms beforehand and those who were sceptical. [25,26] This is the approach used in STAMPEDE. Many instances of early stopping for benefit appear to occur on a random high [27,28] and the credibility and interpretability of a trial can be adversely affected: caution should be applied in this respect.

## Other issues

### Positive perceptions of the MAMS design

The MAMS design has been perceived by some observers to have many complexities above those of the traditional two-arm RCT. The view of groups central to the conduct of the trial, below, has been very positive, with no serious concerns expressed.

#### Clinicians and surgeons

Doctors around the UK have been generally enthusiastic about the questions and methods in STAMPEDE, although some are inevitably more cautious. The recruitment rate has been good, mirroring the experience in other multi-arm trials, which have already recruited at excellent rates[29,30]. Efficient recruitment to STAMPEDE requires good collaboration between urologists and oncologists: the majority of men eligible for STAMPEDE would be routinely seen and managed by urologists, but randomisation to some of the arms requires management by oncologists, particularly those involving cytotoxic chemotherapy with docetaxel. The initiation and function of the multi-disciplinary team (MDT) as part of the UK cancer plan has facilitated this process considerably, as it has in other non-MAMS trials.

#### Men with prostate cancer

Men with prostate cancer have shown a real willingness to participate in STAMPEDE. The Trial Management Group (TMG) has two patient members whose input has been invaluable. Informed consent is taken from all participating patients. With six trial arms, a major issue is how to provide the patient with sufficient information without overload. The trial addresses this by using a two-part patient information sheet (PIS) where the information about the trial given before randomisation includes only summary information on the individual trial arms; specific details of the allocated therapy are given after randomisation. If interested, men may request all of the arm-specific information sheets at any time before randomisation. In this way, men can determine the level of written information they would like before joining the trial. This has been positively received judging by feedback from sites and recruitment rates. This approach was approved by the main Research Ethics Committee for the trial. We note that the National Research Ethics Service now routinely recommends the use of a two-part PIS[31], although the emphasis is somewhat different to that used in STAMPEDE. We also note that the PIS is commonly used only as a secondary source of information, supporting the discussions the patient has with the local investigator's team.

#### Funding bodies

STAMPEDE has been approved and partially funded by Cancer Research UK, a major UK cancer charity, with the allocation of grant support through open competition at their Clinical Trials Advisory and Award Committee (CTAAC). The Medical Research Council (MRC) Clinical Trials Unit has agreed to the use of core funding to support central trial staff and MRC has agreed to Sponsor the trial and funding is in place from these bodies to cover all stages of the trial.

#### Pharmaceutical industry partners

The three industry partners for STAMPEDE (Novartis, Sanofi-Aventis and Pfizer) have all been supportive and have provided important input at various stages, including the provision of trial drug and modest educational grants. Developing agreements between academic and industry partners is often a rate-limiting step for clinical trials. Synchronous negotiations with three companies provided some additional complexities and delays because of administrative issues, but this is not unique to MAMS trials: many traditional two arm trials require agreements with more than one industry partner eg if a trial arm is assessing combination treatment.

#### Governance, regulatory and ethical bodies

There have been no significant issues for MAMS trials in this area. STAMPEDE received approval in the "old" UK regulatory system just before the implementation of the EU Clinical Trials Directive (EC 2001/20) in May 2004; this was carried forward to a Clinical Trials Authorisation (CTA) by the Medicines and Healthcare Products Regulatory Authority (MHRA) and recruitment is taking place widely across the UK. A limited number of sites have not been able to participate because of local governance issues. However, these concerns were not about the MAMS design but about local capacity to provide intravenous therapy (zoledronic acid and docetaxel) to this patient group, who had not routinely received such treatment previously.

### Why STAMPEDE is not designed as a factorial trial

A factorial trial design may sometimes be used to simultaneously address more than one research comparison in the same trial population but factorial trials are typically designed with low power for estimating interactions between treatments and are weakened if there is any interaction between the treatments. Relatively little is known about most of the agents assessed in STAMPEDE when used in combination with hormone therapy and with each other. Thus, there is no good evidence to support the notion that the treatment effects would only be additive at the patient level and we cannot rule out other forms of interaction: synergy, antagonism and a ceiling effect. Synergy occurs if the effectiveness of X+Y+Z were much greater than the effectiveness of X+Y plus X+Z. This has been observed *in vitro *with other taxanes[32]. A factorial design could lead to overestimation the individual effectiveness of Y and Z. Alternatively, antagonism occurs if the effectiveness of X+Y+Z were much less than the effectiveness of X+Y plus X+Z. Such a factorial design could underestimate the individual effects of acidy and Z. Finally, a ceiling effect could limit the potential activity that these various classes of agents can add to the control arm eg the effectiveness of X+Y+Z would be similar to X+Y and to X+Z.

Given that a factorial design might over- or under-estimate the individual effects of docetaxel, zoledronic acid and celecoxib, STAMPEDE has not been designed in a factorial way, instead comparing each research arm directly against the control arm.

### How many arms a MAMS trial might have

STAMPEDE is a 6-arm trial, but the MAMS design may be used for trials with different numbers of arms and also with 2 arms. However, the efficiencies may become more pronounced when 4 or more arms are used. The upper limit for the number of arms would likely be determined by practical issues rather than those of statistical design; there is no reason why, for example, a trial of 9 research arms and a control arm may not be feasible, particularly if the intermediate outcome measure was a very early measure of activity.

### Adding new arms during the trial

Additional arms could have been included from the outset of the trial, but for practical reasons we chose just five research arms. Each of these had a sound rationale for formal testing and the industry partners were willing to collaborate. It may also be possible to add in additional arms at a later stage using a MAMS design. This possibility has started to be explored in STAMPEDE when discussions were held with a further pharmaceutical company about including a potentially interesting new agent. We used that opportunity to identify the practical and financial issues which we should need to overcome in order to add further arms. Importantly, new research arms could only be compared against control arm patients randomised after the new research arm was added. Any research arms added later on would need to undergo the same assessments and to pass the same activity hurdles as the original arms. Inevitably, any assessments of new research arms would be offset in time compared to the original arms.

## Conclusion

The MAMS design allows researchers to assess multiple therapeutic approaches simultaneously by using an intermediate outcome measure to focus resources on those treatment options showing early evidence of positive activity. This design increases the chance of a single trial providing a positive result and saves time and money compared to separate sequential trials. This design will become more commonly used and a further MAMS trial, ICON6, has now been launched for women with ovarian cancer [33].

We have used STAMPEDE to illustrate the practical implementation of the MAMS trial design. STAMPEDE is the first to address many of the practical issues that arise by using MAMS methodology. Future MAMS trials may face other issues, for example if the intermediate outcome measure is binary. Recruitment to STAMPEDE is ongoing and the main results are not expected to be known until around 2013. STAMPEDE has shown that this design is acceptable to patients, researchers, funding bodies, industry and sponsoring organisations and that MAMS trials can be successfully undertaken.

## Abbreviations

ADT: Androgen deprivation therapy; CTA: Clinical Trials Authorisation; CTAAC: Clinical Trials Advisory and Award Committee; CTU: MRC Clinical Trials Unit; EU: European Union; FFS: Failure-free survival; HR: Hazard ratio; IDMC: Independent Data Monitoring Committee; MAMS: Multi-Arm Multi-Stage; MHRA: Medicines and Healthcare Products Regulatory Authority; MRC: Medical Research Council; OM: Outcome measure; OS: Overall survival; PIS: Patient Information Sheet; RCT: Randomised Controlled Trial; STAMPEDE: Systemic Therapy for Advanced or Metastatic Prostate cancer: Evaluation of Drug Efficacy; TMG: Trial Management Group; TSC: Trial Steering Committee.

## Competing interests

Matthew Sydes, Mahesh Parmar, Rachel Morgan, Karen Sanders and Patrick Royston are all employed by the Medical Research Council, a publicly-funded body in the UK, who are sponsoring the trial. The other author(s) declare that they have no competing interests.

## Authors' contributions

MRS led the drafting of the manuscript, participated in the design and implementation of the trial. MKBP co-led the drafting of the manuscript, participated in the design and implementation of the trial and developed MAMS methodology and program used for trial sample size. NDJ, NWC, MDM and DPD helped to draft the manuscript and participated in the design and implementation of the trial. RCM helped to draft the manuscript and participated in the implementation of the trial. KS helped to draft the manuscript and participated in the implementation of the trial. PR helped to draft the manuscript and developed MAMS methodology and program used for trial sample size. All authors have read and approved the final manuscript.

## Authors' information

Matthew R Sydes is the trial statistician and project lead. Mahesh KB Parmar is the Programme Leader at MRC CTU and Head of MRC CTU Cancer Group. Nicholas D James is the Chief Investigator and is Chair of the Trial Management Group. Noel W Clarke is the Vice-chair of TMG and current Chair of the NCRI prostate Clinical Studies Group. David P Dearnaley is a clinical member of TMG and previous Chair of the NCRI prostate Clinical Studies Group. Malcolm D Mason is the Vice-chair of TMG. Rachel C Morgan was the assistant statistician. Karen Sanders is the Trial Manager.
